# Angiotensin Converting Enzyme 2, Angiotensin-(1-7), and Receptor Mas Axis in the Kidney

**DOI:** 10.1155/2012/414128

**Published:** 2012-02-08

**Authors:** Sergio Veloso Brant Pinheiro, Ana Cristina Simões e Silva

**Affiliations:** Pediatric Nephrology Unit, Department of Pediatrics, Federal University of Minas Gerais (UFMG), 30130-100 Belo Horizonte MG, Brazil

## Abstract

In the past few years the understanding of the renin-angiotensin system (RAS) has improved, helping to better define the role of this system in physiological conditions and in human diseases. Besides Angiotensin (Ang) II, the biological importance of other Ang fragments was progressively evidenced. In this regard, Angiotensin- (Ang-) (1-7) was recognized as a biologically active product of the RAS cascade with a specific receptor, the G-protein-coupled receptor Mas, and that is mainly formed by the action of the angiotensin-converting enzyme (ACE) homolog enzyme, ACE2, which converts Ang II into Ang-(1-7). Taking into account the biological effects of these two mediators, Ang II and Ang-(1-7), the RAS can be envisioned as a dual function system in which the vasoconstrictor/proliferative or vasodilator/antiproliferative actions are primarily driven by the balance between Ang II and Ang-(1-7), respectively. In this paper, we will discuss our current understanding of the ACE2/Ang-(1-7)/Mas axis of the RAS in renal physiology and in the pathogenesis of primary hypertension and chronic kidney disease.

## 1. Introduction

### 1.1. Historical Background of the ACE2/Ang-(1-7)/Mas Axis of the RAS

In the past few years the understanding of the renin-angiotensin system (RAS) has improved, helping to better define the role of this system in physiological conditions and in human diseases. Following the seminal study of Schiavone and coworkers [1] demonstrating that Angiotensin- (Ang-) (1-7) is a biologically active peptide of the RAS, several reports have clearly shown that this heptapeptide plays important functions in cardiovascular and renal system [2, 3].

The identification of the angiotensin-converting enzyme (ACE) homologue, ACE2, as the main Ang-(1-7)-forming enzyme was essential to establish a preferential enzymatic pathway for the production of this angiotensin peptide [4, 5]. ACE2 can cleave Ang I to form Ang-(1-9) [4], which is subsequently converted to Ang-(1-7) through ACE and neutral-endopeptidase 24.11 (NEP) activity [6]. However, the main substrate for ACE2 is Ang II, which is converted into Ang-(1-7) [7]. Consequently, ACE2 plays a pivotal role in the balance between both RAS mediators, Ang II and Ang-(1-7), once this enzyme can convert Ang II, a vasoconstrictor peptide, into Ang-(1-7), a vasodilator peptide. However, it should be mentioned that, besides ACE2, other enzymes might contribute to Ang-(1-7) formation such as prolylendopeptidase (PEP), prolylcarboxypeptidase (PCP), and NEP [8–10].

Further support for the relevance of Ang-(1-7) was achieved with the description of the orphan receptor Mas as a functional ligand site for this angiotensin [11]. This discovery was a confirmation of results previously obtained with the Ang-(1-7) antagonists, suggesting that Ang-(1-7) exerted its actions through a specific receptor, distinct from Ang II receptors type 1 (AT_1_) and type 2 (AT_2_) [12, 13].

It is now conceived that the RAS axis formed by ACE2, Ang-(1-7), and Mas is able to counter balance many of the well-established actions of the ACE-Ang II-AT_1_ receptor axis [2, 3, 14, 15]. Accordingly, the activation of the vasodilator/antiproliferative axis might represent an endogenous protective mechanism against the deleterious effects elicited by the ACE-Ang II-AT_1_ receptor axis, especially in pathological conditions [2, 3, 14]. However, the role of ACE2-Ang-(1-7)-Mas axis appears to go far beyond a counterregulatory action. 

This paper will briefly highlight recent findings concerning the renal effects of the ACE2-Ang-(1-7)-Mas axis in renal physiology and discuss its potential role in disease states.

### 1.2. The Role of ACE2/Ang-(1-7)/Mas Axis in Renal Physiology

A growing body of evidence supports the relevance of Ang-(1-7) for the regulation of renal function. Ang-(1-7) is present in the kidney at concentrations that are comparable to Ang II [8, 15]. The processing pathways for Ang-(1-7) in the circulation and kidney appear to be distinct. In the circulation, NEP is one of the major enzymes that produce Ang-(1-7) from Ang I or Ang-(1-9) [8]. In the kidney, NEP may contribute to both the synthesis as well as the degradation of Ang-(1-7). This enzyme cleaves Ang I to Ang-(1-7) and also metabolizes the peptide at Tyr^4^-Ile^5^ bond to form Ang-(1-4) and Ang-(5-7) [16, 17]. ACE2 seems to be the primarily responsible for Ang-(1-7) synthesis in the renal tissue [15]. 

It should be pointed that there are gender differences in renal activity of ACE2 and in the mRNA expression for this enzyme at renal tissue. In this regard, Ji and coworkers showed that ovariectomy decreased ACE2 protein (30%) and mRNA expression (36%) in renal wrap hypertension in rats, while 17-beta-estradiol replacement prevented these effects [18]. In addition, the infusion of Ang-(1-7) attenuated renal injury which was exacerbated by ovariectomy in this experimental model [18]. The authors concluded that 17-beta-estradiol-mediated upregulation of renal ACE2 and the consequent increased Ang-(1-7) production might protect against hypertensive renal disease. More recently, Liu and coworkers found that ACE2 activity was higher in the kidney of male mice compared to the kidney of females [19]. These authors believe that sex differences in renal ACE2 activity in intact mice are due, at least in part, to the presence of 17-beta-estradiol in the ovarian hormone milieu and not to the testicular milieu or to differences in sex chromosome dosage (2X versus 1X; 0Y versus 1Y) [19]. Therefore, the regulation of renal ACE2 by 17-beta-estradiol has particular implications for women across their life span since this hormone changes radically during puberty, pregnancy, and menopause.

Ang-(1-7) is the main product obtained in preparations of isolated proximal tubules and exists in urine at higher concentrations than Ang II [17]. The heptapeptide is also present in the distal convoluted tubules and collecting ducts [20]. Chappell et al. [15] demonstrated that the distribution of ACE2 within renal tubules is similar to that of Ang-(1-7). This finding was a preliminary evidence for the direct conversion of Ang II to Ang-(1-7) in the kidney. In keeping with these observations, Ferrario et al. [21] supported a role for ACE2 in Ang-(1-7) formation from Ang II in the kidney of normotensive rats. This study showed an increased ACE2 activity measured in renal tissue of rats given either lisinopril or losartan [21]. These data further suggest that increased levels of Ang-(1-7) in the urine of animals under ACE inhibition or AT_1_ receptor blockade might reflect an intrarenal formation of this heptapeptide [21].

Many studies have addressed the complexity of renal actions of Ang-(1-7) [8, 15, 22–26]. Differences between species, local and systemic concentrations of Ang-(1-7), nephron segment, level of RAS activation, and sodium and water status can be responsible for discrepant results concerning renal effects of Ang-(1-7). A diuretic/natriuretic action of Ang-(1-7) has been described in several *in vitro* [27–30] and *in vivo* experimental models, mostly by inhibition of sodium reabsorption at proximal tubule [28, 31, 32]. Ang-(1-7) seems to be a potent inhibitor of Na-K-ATPase activity in the renal cortex [33] and in isolated convoluted proximal tubules [34]. In renal tubular epithelial cells, Ang-(1-7) inhibited transcellular flux of sodium, which was associated with activation of phospholipase A_2_ [27]. *In vitro* studies also indicated that Ang-(1-7) modulates the stimulatory effect of Ang II on the Na-ATPase activity in proximal tubule through an A779-sensitive receptor. In this regard, Bürgelová et al. [34] showed that intrarenal administration of Ang-(1-7) produced natriuresis and blocked the antinatriuretic actions of Ang II.

On the other hand, our group and other investigators have observed an antidiuretic/antinatriuretic effect induced by Ang-(1-7), especially in water-loaded animals [11, 22, 23, 32, 35–41]. Ang-(1-7) has a potent antidiuretic activity in water-loaded rats [38, 39] and mice [35] probably mediated by the receptor Mas [11]. *In vitro*, Ang-(1-7) increased the water transport in the inner medullary collecting duct through an interaction between receptor Mas and the vasopressin type 2 receptor with subsequent adenylate cyclase activation [41]. These data were in accordance with the renal effects produced by the selective Ang-(1-7) receptor Mas antagonists, the compounds A-779 [12, 38–41] and D Pro^7^-Ang-(1-7) [42]. The administration of these antagonists exerts a diuretic effect associated with an increase in glomerular filtration rate and in water excretion [38–42]. These findings suggest that endogenous Ang-(1-7) takes part in the regulation of glomerular filtration and of water handling at renal level.

The physiological relevance of Ang-(1-7) was further corroborated by the demonstration that Ang-(1-7) is an endogenous ligand for the G-protein-coupled receptor Mas in the kidney [11]. Immunocytochemical data reveal a similar distribution for Ang-(1-7), ACE2, and the Mas receptor within the tubular epithelium of the kidney [15]. Experimental data obtained with receptor Mas agonists and antagonists help understanding the role of this receptor in renal physiology. In water-loaded C57BL/6 mice, the administration of the oral agonist of receptor Mas, the compound AVE 0991, produced a significant reduction in urinary volume, associated with an increase in urinary osmolality [35]. The receptor Mas antagonist, A-779, completely blocked the antidiuretic effect of AVE 0991 [35]. As observed previously for Ang-(1-7) [11], the antidiuretic effect of AVE 0991 after water load was blunted in mice with genetic deletion of receptor Mas [35]. *In vitro* receptor autoradiography in C57BL/6 mice showed that the specific binding of ^125^I-Ang-(1-7) to mouse kidney slices was displaced by AVE 0991, whereas no effects were observed in the binding of ^125^I-Ang II or ^125^I-Ang IV [35]. More recently, these findings were further corroborated taking advantage of a novel transgenic rat model, TGR(A1-7)3292, that expresses an Ang-(1-7)-producing fusion protein which produces chronic elevation in Ang-(1-7) plasma concentration [43]. In this study, transgenic rats presented a significant reduction of basal urinary volume and of free water clearance, without changing plasma levels of vasopressin and the mRNA expression of Mas and vasopressin type 2 receptors in renal tissue [43].

Beside important tubular actions, Ang-(1-7) also contributes to renal hemodynamic regulation. The ability of the kidney to generate high intratubular and interstitial concentrations of Ang II and Ang-(1-7) allows the kidney to regulate intrarenal levels of these angiotensins in accord with the homeostatic needs for the regulation of renal hemodynamics, tubular reabsorption, and sodium balance. When the RAS is inappropriately stimulated, high intrarenal Ang II levels, acting on AT_1_ receptors, can lead to both systemic and glomerular capillary hypertension, which can induce hemodynamic injury to the vascular endothelium and glomerulus [44, 45]. In addition, direct profibrotic and proinflammatory actions of Ang II may also promote kidney damage [44–46]. On the other hand, Ren et al. [47] reported that Ang-(1-7)-induced dilatation of pre-constricted renal afferent arterioles in rabbits and Sampaio et al. [48] showed that an infusion of low concentrations of Ang-(1-7) increased renal blood flow in rats. Ang-(1-7) also attenuated the effect of Ang-II-induced pressor responses and Ang-II-enhanced noradrenaline release to renal nerve stimulation in rat isolated kidney [49]. These results opened the possibility that Ang-(1-7) can also act as a physiological regulator of intraglomerular pressure, probably opposing the hypertensive and fibrogenic effects of Ang II.

## 2. The Role of ACE2/Ang-(1-7)/Mas Axis in Renal Diseases

### 2.1. Current Experimental Evidence

Experimental studies have also indicated a role for the Ang-(1-7)-Mas interaction in the regulation of matrix proteins deposition in the heart and liver [50, 51]. Our group has shown fibronectin and collagen III deposition in the kidney of mice with genetic deletion of receptor Mas, suggesting that these genetic modified animals exhibit a phenotype predisposition to renal fibrosis [52]. Accordingly, at initial stages of collagen deposition and renal fibrosis, type III collagen appears in greater amounts than do type I. As renal fibrosis progresses, there is a proportional decrease in type III collagen, and tubulointerstitial fibroblasts secrete collagen types I, III, IV, and V in response to TGF-beta, epidermal growth factor, and interleukin-2 [53, 54]. More recently, Zhang et al. demonstrated that infusion of angiotensin-(1-7) reduces glomerulosclerosis through counteracting angiotensin II in experimental glomerulonephritis [55], suggesting that Ang-(1-7) is also relevant for modulating renal fibrosis in disease states.

Although a protective role for Ang-(1-7) in renal fibrosis remains speculative, our findings in animals with genetic deletion of receptor Mas support this hypothesis [52]. In addition, many studies have shown that Ang-(1-7) exerts inhibitory effects on vascular and cellular growth mechanisms. The molecular mechanisms for the antiproliferative action of Ang-(1-7) include the stimulation of prostaglandin and cAMP production as well the inhibition of mitogen-activated protein (MAP) kinases [56]. The antiproliferative effects of Ang-(1-7) in vascular smooth muscle cells [57], liver tissue [51], and cardiomyocytes [58] seem to be mediated by receptor Mas. Moreover, Mas-deficient mice exhibited an impairment of heart function associated with changes in collagen expression toward a profibrotic profile [50]. Gallagher and Tallant [59] also reported the inhibition of human lung cell growth by Ang-(1-7) through a reduction in the serum-stimulated phosphorylation of extracellular signal-regulated kinase (ERK) 1 and ERK2. As the ERK cascade is activated in response to different stimuli, such as growth factors, cytokines, or DNA-damaging agents, the stimulation of the ACE2-Ang-(1-7)-Mas axis could be effective in halting glomerulosclerosis. Su et al. [60] have reported that Ang-(1-7) inhibits Ang II-stimulated MAP kinases phosphorylation in proximal tubular cells. Thus, the generation of Ang-(1-7) by proximal tubular ACE2 could counteract the proliferative effects of locally produced Ang II [60]. 

In keeping with this possibility, recent studies suggested a protective role for ACE2 in the kidney. Kidney diseases have been associated with a reduction in renal ACE2 expression, possibly facilitating the damaging effects of Ang II. Acquired or genetic ACE2 deficiency also appears to exacerbate renal damage and albuminuria in experimental models, supporting this hypothesis [61–67]. In addition, chronic blockade of ACE2 with the enzyme inhibitor MLN-4760 in control or diabetic mice produced albuminuria and matrix protein deposition [65]. More recently, Dilauro et al. [66] showed that ACE2 is downregulated in the renal cortex of mice that underwent subtotal nephrectomy. The reduction of renal ACE2 in nephrectomized animals led to proteinuria via an AT_1_ receptor dependent mechanism [66]. Accordingly, the renal expression of ACE2 was also reduced in an experimental model of renal ischemia/ reperfusion [67]. Taken together, these findings suggest that decreased ACE2 activity may be involved in the pathogenesis of kidney disease, possibly by disrupting the metabolism of angiotensin peptides [68]. Taking into account the enzymatic properties of the two ACEs and of the two main mediators Ang II and Ang-(1-7), the RAS can be envisioned as a dual function system in which the vasoconstrictor/proliferative or vasodilator/antiproliferative actions are primarily driven by the ACE–ACE2 balance. Accordingly, an increased ACE/ACE2 activity ratio will lead to increased Ang II generation and increased catabolism of Ang-(1-7), favoring vasoconstriction, while a decreased ratio will decrease Ang II and increase Ang-(1-7) levels, facilitating vasodilatation [68–70]. 

On the other hand, some studies pointed to a deleterious role for Ang-(1-7) at renal system. For instance, the study of Esteban and coworkers using mice with genetic deletion of receptor Mas showed very discrepant results in relation to renal function when compared to our findings [71]. While our research group [52] showed that the genetic deletion of receptor Mas in C57Bl/6 mice led to glomerular hyperfiltration, proteinuria and renal fibrosis, Esteban et al. [28] reported that renal deficiency of Mas diminished renal damage in unilateral ureteral obstruction and in ischemia/reperfusion injury, and that the infusion of Ang-(1-7) to wild-type mice elicited an inflammatory response. Furthermore, animal models of renal diseases have also showed discrepant findings. Zhang et al. [55] showed that a 5-day infusion of Ang-(1-7) improved glomerulosclerosis in a rat model of thy-1-induced glomerulonephritis, whereas Velkoska et al. [72] verified that a 10-day infusion of the same concentration of Ang-(1-7) in rats with subtotal nephrectomy was associated with deleterious effects on blood pressure and the heart, with increase in cardiac ACE, and decrease in cardiac ACE2 activity. Although these findings are conflicting, cell-specific signaling pathways associated with Ang-(1-7) in the kidney could play a role in the variable response. In this regard, in the proximal tubule Ang-(1-7) displays growth inhibitory properties and antagonizes the effects of Ang II [60], whereas in mesangial cells, it appears to stimulate cell growth pathways [73]. In addition, the vascular and tubular effects of Ang-(1-7) in the kidney appear to be importantly influenced by experimental conditions and the level of RAS activation [74].

### 2.2. Current Clinical Evidence

Agonists and antagonists of the Ang-(1-7)-Mas axis probably possess a therapeutic potential for the modulation of sodium and water excretion in many physiologic and pathologic renal conditions, such as arterial hypertension, nephrogenic diabetes insipidus, glomerular diseases, chronic kidney disease (CKD), and diabetic nephropathy (see [68–70], for review). Ang-(1-7) can be measured in plasma and urine samples collected in healthy subjects and in patients with diverse clinical conditions (see [68–70], for review). Changes in blood pressure, in blood volume, in sodium intake and in renal function were able to modify the levels of Ang-(1-7) measured in plasma, renal tissue, and urine [75–81]. In addition, the concentration of the heptapeptide may differ in plasma and urine samples of the same subject. Accordingly, Ferrario and coworkers have reported that Ang-(1-7) is excreted in the urine of normal healthy adult volunteers in amounts 2.5-fold higher than those measured in plasma [82]. In the same study, it was also observed that untreated adults with primary hypertension exhibited a lower urinary excretion of Ang-(1-7) than normotensive controls and urinary concentrations of Ang-(1-7) were inversely correlated with blood pressure [82]. 

In pediatric patients, Simões e Silva and coworkers have reported significant differences among circulating Ang II and Ang-(1-7) levels in renovascular disease and in primary hypertension [83]. Children with renovascular hypertension had plasma levels of Ang II higher than of Ang-(1-7) and the successful correction of unilateral renal artery stenosis produced a return of circulating angiotensins to levels similar to those in healthy subjects. In contrast, patients with primary hypertension had significant elevation of circulating Ang-(1-7), while the levels of Ang I and Ang II were within the same range as in healthy subjects. In addition, the achievement of blood pressure control with calcium channel blockers did not change plasma concentration of Ang-(1-7) and Ang II. The physiopathological meaning of increased levels of only Ang-(1-7) in pediatric primary hypertension is still unknown and raises the question whether this elevation is a compensatory mechanism that opposes deleterious renal and cardiovascular effects of Ang II or whether, at supra physiological concentrations, Ang-(1-7) could act as another RAS mediator of renal dysfunction.

In addition, Simões e Silva et al. have demonstrated a significant increase in plasma Ang-(1-7) and Ang II levels among hypertensive children with CKD stage III when compared to normotensive CKD patients with the same stage of renal dysfunction [76]. While the presence of hypertension affected plasma concentration of both peptides, the progression to end stage renal disease was accompanied by more pronounced elevation only in Ang-(1-7) levels. Ang II levels were similarly elevated despite the level of renal dysfunction. Taken together, these data support a preferential production of Ang-(1-7) in end stage renal disease [76]. Future studies are needed to elucidate the physiopathological role of this heptapeptide in human CKD.

Another important aspect to be considered is the elevation of plasma Ang-(1-7) during chronic RAS inhibition [76–79]. The renoprotective actions of ACE inhibitors and AT_1_ receptor blockers clearly involve multiple pathways including antiproliferative and antifibrogenic effects [78, 80, 81]. In particular, an altered balance between Ang II and Ang-(1-7) might be related to the mechanism of action of ACE inhibition and AT_1_ receptor blockade. Studying healthy subjects, Kocks et al. [79] showed that, during ACE inhibition, the administration of a low sodium diet did not affect plasma levels of Ang II but induced a significant elevation in Ang-(1-7) concentration. Consequently, the combination of ACE inhibition and a low sodium diet appeared to shift the balance between Ang-(1-7) and Ang II towards Ang-(1-7), which in turn might contribute to the therapeutic benefits of ACE inhibition [79].

## 3. Concluding Remarks

**Figure 1:**
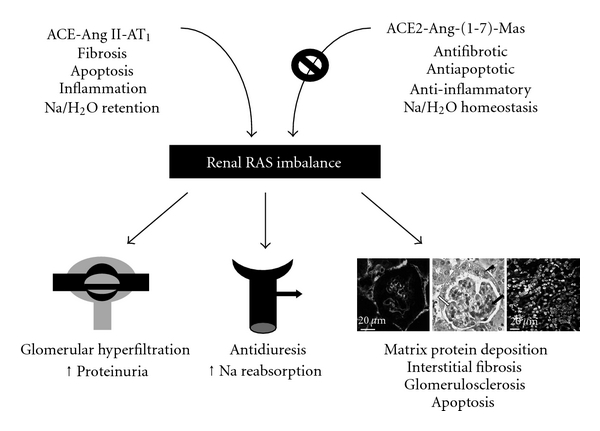
Proposed mechanisms for the role of ACE-Ang II-AT_1_ receptor axis in excess of ACE2-Ang-(1-7)-Mas receptor axis at renal level.

The current evidence supports the existence of a counterregulatory axis within the RAS formed mainly by the ACE2-Ang-(1-7)-receptor Mas axis. The primary function of this axis is to oppose the effects of the major component of the RAS, Ang II. Experimental and clinical studies have demonstrated a role for the ACE2/Ang-(1-7)/Mas axis in the regulation of renal function, in arterial hypertension, and in the progression of CKD.  Figure 1 shows the proposed mechanisms for the role of ACE-Ang II-AT_1_ receptor axis in excess of ACE2-Ang-(1-7)-Mas receptor axis at renal level. Therefore, the disproportion between both RAS axes might represent an important pathway for CKD progression (Figure 1). Further research on the contribution of the ACE2/Ang-(1-7)/Mas axis to renal pathophysiology should lead to the development of new pharmacologic approaches resulting in the design of molecular or genetic means to increase the expression of ACE2, allow for increased tissue levels of Ang-(1-7), or both.
